# Patient safety: knowledge, influence and attitude among physicians: an exploratory study

**DOI:** 10.1186/s42506-019-0022-9

**Published:** 2019-09-04

**Authors:** Noha Asem, Hend Aly Sabry, Eman Elfar

**Affiliations:** Public Health and Community Medicine Department, Faculty of Medicine, Cairo University, Kasr Al Ainy St, Cairo, 11956 Egypt

**Keywords:** Patient safety, Knowledge, Influence, Attitude, Physician

## Abstract

**Background:**

Patient safety is regarded as a global problem by which both developed and developing worlds are affected. It is defined as avoidance and prevention of patient injuries or adverse events, which could result during health care delivery. This study aimed to identify physicians’ knowledge, influence, and attitude toward patient’s safety in the faculty of medicine, Cairo university.

**Materials and methods:**

A cross-sectional study was conducted on 187 postgraduate physicians of different specialties working in the faculty of medicine, Cairo University. Anonymous self-administered questionnaires were distributed. The questionnaire is one of a series of tools designed for evaluation of the pilot implementation of the World Health Organization patient safety curriculum for medical schools.

**Results:**

Calculated attitude score was relatively higher than influence, then knowledge score (median scores were 4.25, 3.1, and 2.5 respectively). There was no difference in knowledge, attitude, and influence scores by different personal characteristics as gender, specialty, workplace, graduation year except for higher influence score among physician who received inpatient safety training (*p* = 0.016). There was a weak positive significant correlation between knowledge and influence scores and between influence and attitude scores (*r* = 0.25, *p* = 0.002; *r* = 0.27, *p* < 0.001 respectively).

**Conclusion:**

Higher patient safety positive attitude than influence and knowledge is pinpointed in physicians of different specialties in the faculty of medicine, Cairo University. This raises the attention to the importance of implementation of continuing patient safety education programs.

## Introduction

Patient safety is an issue that has increasingly gained the attention of the whole world [1]. It is defined as avoidance and prevention (or amelioration) of patient injuries or adverse events, which could result during health care delivery [2]. Since the release of the “To Err is human: Building a Safer Health System” report (1999) by the Institute of Medicine (IOM), patient safety has become one of the priorities of many health care systems. According to this report, almost 98,000 die in United States (US) hospitals every year as a result of preventable medical errors. Consequently, the occurrence of medical errors was highly considered by health policy-makers and stakeholders worldwide [1].

From the well-known causes of errors in an organization that may lead to adverse events are lack of or miscommunication, lack of following safety procedures, inadequate supervision, corruption in continuity of care, excessive workload, insufficient staff numbers plus, and fatigue of healthcare providers [3]. The adverse event is that an unintended injury that actually results in temporary or permanent disability or death due to management during healthcare not due to an underlying disease. To determine that it is preventable, we need to ascertain that a process failure happened [4]. The majority of these adverse events were believed to stem from a complex chain of events rather than individual human errors. The IOM report delivered the message that we must focus on system failures before human factors if we want to prevent the occurrence of medical errors [1].

Reaching this point, it was agreed that to prevent these errors, we must first understand how they happen which actually starts by their reporting. The IOM report recommended the development of non-punitive environments in hospitals, which promote incident reporting. The importance of reporting and analysis of near-miss data was thus intensified [1]. This same report stated that the biggest challenge to moving toward a safer health system is changing the culture from blaming individuals for errors to handling errors as improvement opportunities for the system. Both reporting of errors and their disclosure affect the culture of safety. Consequently, patient safety is built in healthcare culture if the means by which we identify, report, and communicate errors to those involved is accepted [5].

The safety culture is the shared values, attitudes, perceptions, and ways of behavior among the individuals in the organization that determine the commitment of all members to act toward ameliorating patient harm which may result during care delivery [6]. This includes the commitment of the leadership to discuss errors and learn from them through using systems for reporting and analyzing adverse events followed by recognizing workers as heroes who improved safety not villains who committed errors [2]. Analysis of root causes of the problems will not succeed in disclosing latent causes of error if staff, bound by “code of silence,” cannot comfortably expose weaknesses in processes which they are responsible for [7].

Patient safety is regarded as a global problem by which both developed and developing worlds are affected [8]. In the developed world, studies tell that in hospitals, rates of adverse events actually were much higher than we previously thought of, with a figure of at least 8%; 50% of them were assumed to be preventable [4]. On the other hand, in developing countries, there is evidence that the level of awareness among policymakers and providers about the risk of unsafe healthcare is increasing slowly but steadily. In some developing countries, the risk of healthcare-associated infection is found to be about 20 times higher than that found in developed countries. A study performed in the East Mediterranean Region (EMR) showed that adverse events occurred with 18% of inpatient admissions. This same study raised the attention to the increased rate of death and disability, despite high preventability of these adverse events [8].

Encouragement of research to assess the size and nature of the patient safety problem was one of the five specific topics for action passed through resolution WHAA55.18 by the World Health Assembly in year 2002 to urge the World Health Organization (WHO) in order to be more attentive to this important problem. However, most published studies until now came from developed countries.

This knowledge gap seriously hinders our understanding of the extent of the problem mainly at these specific countries. Moreover, it is well known that health systems in developing countries are facing severe health challenges mainly due to their scarce resources and poor infrastructure. Therefore, it is very important to understand the extent of the problem in these countries in order to adopt more effective and efficient curative actions [9].

Until now, only few organizations in Egypt have assessed how much their staff culture backs up patient safety [2]. Measures of perceptions and attitudes about components of safety culture among the working personnel in an organization—safety climate—can deliver an important clue for the level of its safety culture and are particularly informative than observational data. Research has documented the variation in safety climate among variable work areas both across and within institutions. A better disclosure of this point would facilitate the planning and implementation of interventions [6]. Therefore, it becomes a must to identify both the positive and negative perceptions and attitudes of the providers toward the safety environment that may foster or hinder safe patient care [5]. Moreover, organizations are required to deal with providers who may create a negative culture [10].

Many providers claim that they do not have opportunity to join staff development programs related to patient safety applications or to improve their clinical capabilities [8]. Therefore, participation of the providers through questionnaire surveys is required to assess their perceptions of procedures and behaviors in their work that affect safety climate [11]. These surveys also can be used to raise their awareness about this important issue and monitor progress over time [5]. These surveys identify the root causes of the problem, and thereafter advocate at policymakers to develop interventions [8]. In Egypt, although different studies in the patient safety field were carried out, yet handling the issue of “healthcare provider’s perception” was not adequately tackled or been focused on. The objectives of this study are to identify physicians’ knowledge, influence, and attitude toward patient’s safety and to identify factors related to the level of physicians’ knowledge, influence, and attitude.

## Methods

### Study design

This is a cross-sectional observational study among physicians from different specialties.

### Study setting

This study was conducted at the Faculty of Medicine, Cairo university, Egypt.

### Study population

Postgraduate physicians enrolled in Masters’ Program of different specialties in the faculty of medicine, Cairo University.

### Study sample

A purposive sample was taken from physicians attending a training course in research methodology and statistics as a prerequisite for their Masters’ program, academic year 2017/2018. Sample size was calculated by epi info 7 software (Atlanta, Georgia, USA). According to a systematic literature review performed among healthcare staff, the level of knowledge and attitude about patient safety has a very wide variation [12]. Accordingly, expected frequency was set at 50 % and confidence limits were set at 5%; this resulted in sample size estimates of 200 participants for 85% power of the study. Ten percent were added to compensate for dropouts or incomplete responses. Thus, a total of 220 physicians were recruited for the study.

### Study tool

Anonymous self-administered questionnaires were distributed among physicians. The questionnaire is one of a series of tools designed for evaluation of the pilot implementation of the WHO Patient safety curriculum for medical schools. The questionnaire is designed to evaluate medical students’ awareness of patient safety issues and expectations of how patient safety is being managed in the healthcare system[13].

The questionnaire is designed in English and included close-ended 5-point Likert scale questions in which responses consisted of Strongly Agree, Agree, Neutral, Disagree, and Strongly Disagree. Scores for knowledge ranged from (1; low knowledge to 5; high knowledge). The questionnaire includes the following categories: knowledge about errors and safety, safety in the healthcare system, personal influence over safety, personal attitudes to patient safety, and safety at the workplace [13]. Cronbach’s alpha test of reliability was performed for different sections of the questionnaire as well as for the whole questionnaire. Results were as follows: knowledge section = 0.84, influence section = 0.45, attitude section = 0.71, and whole questionnaire = 0.72.

### Pilot study

Before study implementation, a pilot test for the questionnaire form was done among ten potential participants. This was performed to check the validity and clarity of the structured questionnaire as well as to estimate the time needed to complete the questionnaire. Results of the pilot study were excluded from data analysis.

### Administrative considerations

Permission was taken from the coordinator of the course “Research methodology and statistics” before conduction of the study.

### Data analysis

Data was coded, tabulated, and analyzed using SPSS program version 21 for Windows (IBM Corporation, USA, Armonk, New York). Frequency tables were used to describe the data. For Likert scale responses, the answers were coded to numerical values “from 1 to 5.” Value of “1” was given to the wrong answer and value of “5” was given to the most correct answer. Negative statement in the influence section “It is easier to find someone to blame rather than focus on the causes of error” responses were reversed. Data were checked for frequency distribution, skewness, and kurtosis. Kolmogorov–Smirnov test was also performed. This revealed that numerical data were not normally distributed. Accordingly, for inferential statistics, Mann-Whitney *U* test was used to compare between two groups of numerical variables, while Kruskal-Wallis test was used to compare between more than two groups of numerical variables. Spearman’s rho non-parametric correlation was performed among numerical variables. *P* value less than or equal to 0.05 was considered significant.

## Results

The overall response rate to self-administered questionnaire was 85% (187 physicians responded to 220 distributed questionnaires). Age of respondents ranged from 25 to 53 years with mean = 28.8 ± 3.02 years. Females were more than males (59.2%).

The study included physicians from different academic and clinical specialties (medical and surgical). Physicians were from different hospitals. The majority were from Kasr Al-Aini hospitals 96 (51.4 %). Others were from the Ministry of Health and Population (MOHP) hospitals, Educational Institutes, and private hospitals.

Around half of physicians were residents of Cairo and Giza Governorates 95 (50.8%), while others were from different governorates of Upper and Lower Egypt. The majority were graduates of Kasr Al Ainy school of Medicine 122 (65.2%) while others were graduates of other universities in Egypt and outside Egypt. Graduation year ranged from 1988 to 2015. All were postgraduate students enrolled in Masters’ Degree. Thirty six physicians received a patient safety traing courses (20.3 %) (Table 1).

**Table 1:** Personal characteristics of participated physicians in the Faculty of Medicine, Cairo University, 2017/2018

Characteristic	No.	%
Gender		
Male	75	40.8
Female	109	59.2
Specialty		
Surgical	55	30.9
Academic	13	7.3
Medical	110	61.8
Workplace		
University Hospital	100	57.1
Educational Institute	31	17.7
MOHP Hospital	36	20.6
Private	8	4.6
Graduation year		
Within last 5 years	73	41.7
Before last 5 years	102	58.3
Receiving patient safety training courses		
Yes	36	20.3
No	141	79.7

Generally, low level of patient safety knowledge was reported where less than 40% of physicians reported high level of knowledge (score 4 and 5) in all patient safety knowledge items. The main identified knowledge gaps were in ways of speaking up about errors, how to report errors, and the role of healthcare organization in error reporting (high level of knowledge was reported only among 11.8%, 12.4%, and 12.4% of study participants repsectively) (Table 2).

**Table 2:** Knowledge of participated physicians about errors and safety

Knowledge item	1 (low)		2		3		4		5 (high)	
	No.	%	No.	%	No.	%	No.	%	No.	%
Ways of speaking up about error	54	30.2	68	38.0	36	20.1	18	10.1	3	1.7
How to report an error	64	37.2	52	30.2	34	19.8	18	10.5	4	2.3
The role of healthcare organizations in error reporting	59	32.4	54	29.7	47	25.8	19	10.4	3	1.6
Different types of human errors	43	23.2	58	31.4	56	30.3	22	11.9	6	3.2
What would happen if an error is made	47	25.4	68	36.8	33	17.8	26	14.1	11	5.9
Factors contributing to human error	35	18.9	55	29.7	46	24.9	37	20.0	12	6.5
Factors influencing patient safety	18	9.7	44	23.8	57	30.8	49	26.5	17	9.2

With regards to self-perceived influence of participated physicians on error and patient safety, higher influence scores (4 or 5) were reported for ability to talk about their own errors and filling in report forms to improve patient safety (71.5% and 69.9% respectively) while less than one-third (27.9%) reported high influence in ability to ensure that patient safety is not compromised (Table 3).

**Table 3:** Personal influence over safety among participated physicians

Influence item	SD^a^		D^a^		N^a^		A^a^		SA^a^	
	N.	%	N.	%	N.	%	N.	%	N.	%
Able to ensure that patient safety is not compromised	5	2.8	56	31.3	68	38.0	46	25.7	4	2.2
Know how to talk to people who have made an error	3	1.6	46	24.9	78	42.2	47	25.4	11	5.9
Telling Others about an error I made would be easy	16	8.6	53	28.5	50	26.9	55	29.6	12	6.5
Confident about speaking to someone showing a lack of concern to a patient` safety	15	8.2	41	22.5	55	30.2	50	27.5	21	11.5
It is easier to find someone to blame rather than focus on the causes of error	37	20.1	49	26.6	26	14.1	48	26.1	24	13.0
Filing in report forms will help to improve patient safety	6	3.2	23	12.4	27	14.5	74	39.8	56	30.1
Able to talk about my own errors	1	0.5	12	6.5	40	21.5	101	54.3	32	17.2

^a^*SD* strongly disagree, *D* disagree, *N* neutral, *A* agree, *SA* strongly agree

More than three-fourths of participated physicians showed positive attitude toward all error and patient safety attitude statments. Most of them were willing to learn from their mistakes to prevent incidents, appreciate the importance of acknowledging and dealing with their errors, and accepting it as a part of their job (Table 4).

**Table 4:** Personal attitudes to patient safety among participated physicians

Attitude statement	SD^a^		D^a^		N^a^		A^a^		SA^a^	
	No.	%	No.	%	No.	%	No.	%	No.	%
By concerting on the causes of incidents I can contribute to patient safety	1	0.5	10	5.4	36	19.6	104	56.5	33	17.9
If I keep learning from my mistakes, I can prevent incidents	3	1.6	8	4.3	22	11.8	78	41.9	75	40.3
It is important for me to learn how best to acknowledge and deal with my errors by the end of medical school	6	3.2	10	5.4	14	7.5	60	32.3	96	51.6
Acknowledging and dealing with my errors will be an important part of my job	1	0.5	5	2.7	8	4.4	78	42.6	91	49.7

^a^*SD* strongly disagree, *D* disagree, *N* neutral, *A* agree, *SA* strongly agree

**Fig. 1:**
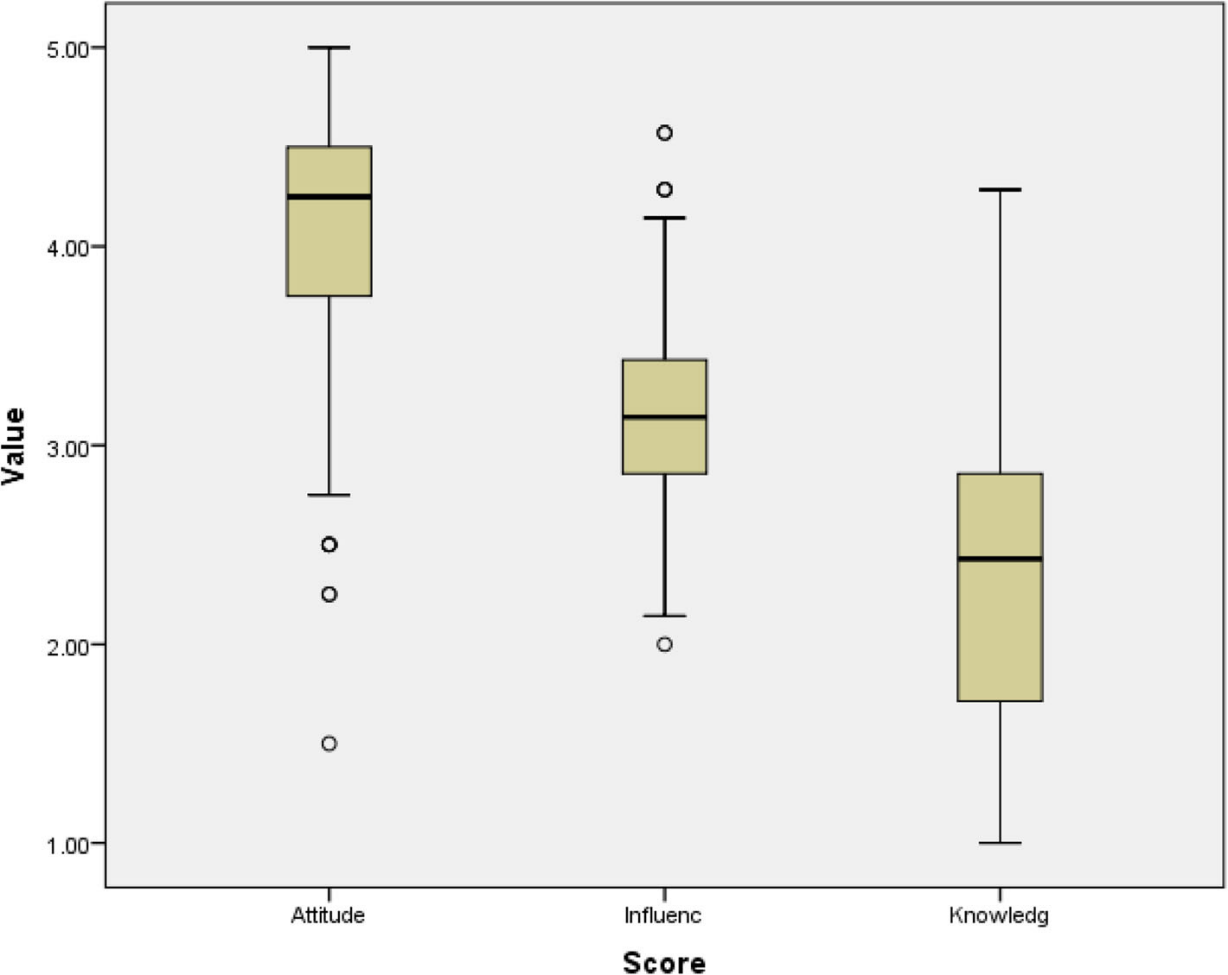
Distribution of the calculated score among participated physician. The text below each box plot indicates calculated score name. Numbers of included participants in each score was 187 physicians. Calculated attitude score was relatively higher than influence, then knowledge score (median scores were 4.25, 3.1, and 2.5 respectively)

Among participated physicians, the calculated attitude score was relatively higher than influence, then knowledge score (median scores were 4.25, 3.1, and 2.5 respectively) (Fig. 1).

There was no difference in knowledge, attitude, and influence scores by different personal characteristics as gender, specialty, workplace, and graduation year except for higher influence scores among physician who took patient safety training courses (*p* = 0.016) (Table 5).

**Table 5:** Relation between personal characteristics and knowledge, influence, and attitude scores among participated physicians

Variable	Knowledge			*P*	Influence			*P*	Attitude			*P*
	Median	IQR			Median	IQR			Median	IQR		
Gender												
Male	2.6	1.7	2.9	0.424	3.1	2.9	3.4	0.362	4.3	3.8	4.8	0.788
Female	2.4	1.7	2.9		3.3	2.9	3.6		4.3	4.0	4.5	
Specialty												
Surgical	2.4	2.0	3.0	0.142	3.3	2.9	3.4	0.169	4.3	3.8	4.5	0.272
Academic	2.0	1.7	2.4		3.0	2.7	3.3		4.3	3.8	4.3	
Medical	2.4	1.7	2.9		3.1	2.9	3.6		4.3	4.0	4.8	
Workplace												
University hospital	2.4	1.9	3.0	0.173	3.1	2.9	3.4	0.633	4.3	3.8	4.5	0.234
Educational institute	2.0	1.3	2.9		3.2	2.9	3.9		4.5	4.3	4.8	
MOH hospital	2.6	2.1	2.9		3.1	3.0	3.4		4.5	4.0	4.8	
Private	2.1	1.7	2.4		3.3	3.1	3.9		4.5	4.4	4.5	
Graduation year												
Within last 5 years	2.6	2.0	3.0	0.055	3.3	2.9	3.4	0.905	4.3	4.0	4.5	0.565
Before last 5 years	2.4	1.6	2.9		3.1	2.9	3.4		4.5	3.8	4.8	
Received patient safety training courses												
Yes	2.4	2.1	3.1	0.257	3.4	3.1	3.7	0.016	4.3	3.9	4.8	0.591
No	2.4	1.7	2.9		3.1	2.9	3.4		4.3	3.8	4.5	

*IQR* interquartile range, MOH Ministry of Health

**Fig. 2:**
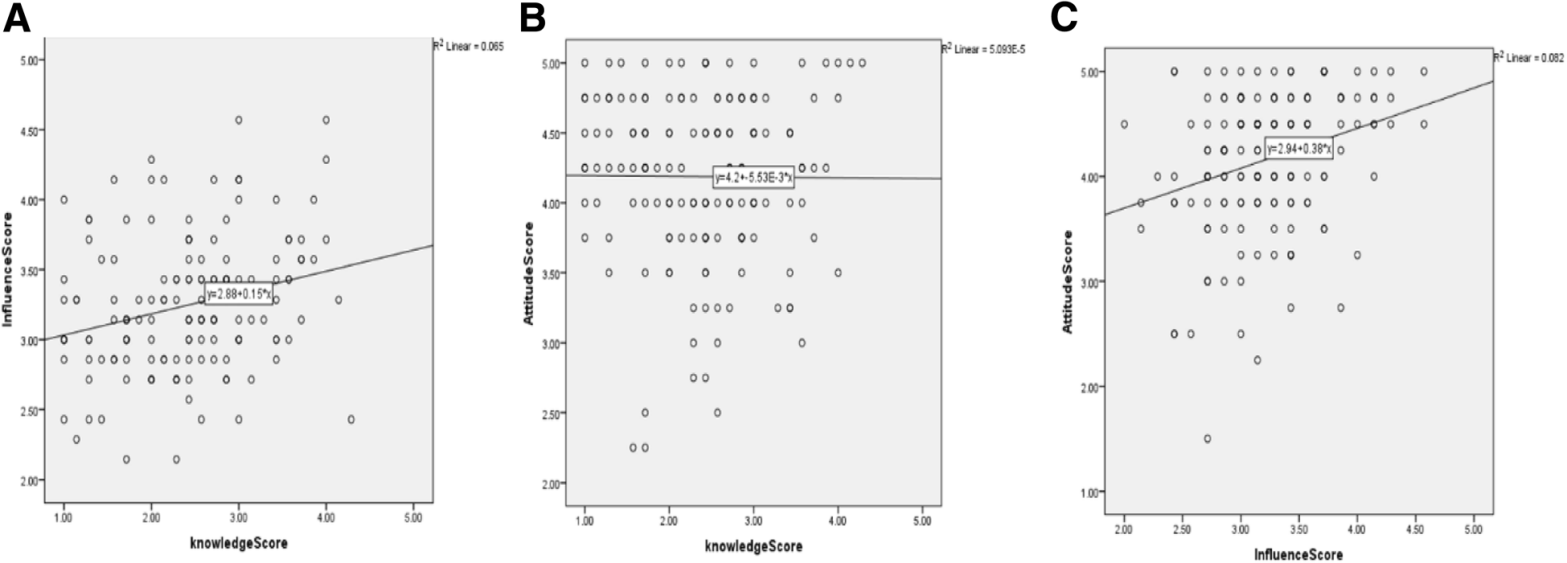
Scatter plots showing correlation between knowledge, attitude, and influence scores among participated physicians. **a** Knowledge score versus influence score. **b** Knowledge score versus attitude score. **c** Influence score versus attitude score. There was a weak positive significant correlation between knowledge and influence scores and between influence and attitude scores (*r* = 0.25, *p* = 0.002; *r* = 0.27, *p* < 0.001 respectively)

There was a weak positive significant correlation between knowledge and influence scores and between influence and attitude scores (*r* = 0.25, *p* = 0.002; *r* = 0.27, *p* < 0.001 respectively) (Fig. 2)

## Discussion

The purpose of this study was to assess the knowledge, influence, and attitudes of medical caregivers toward patient safety and how they are related to each other, in order to have an advanced basis on which to improve their knowledge and attitudes toward that topic. Only a few studies were found from previous literature connected to medical caregivers’ knowledge regarding patient safety. More research has been conducted regarding medical caregivers’ safety attitudes but none on their specific influence. Most of the other studies assessed composites of prevailing patient safety culture [2, 5] or the frequency and nature of adverse events in hospitals [4]. Thus, there exists a gap in the available information as to how knowledge and attitudes regarding patient safety are connected.

“Patient safety” is a relatively new field in Egypt. In a study assessing the perceptions of patient safety culture among health-care workers in Beni-Suef University hospital, it was found that healthcare workers had low perceptions about patient safety culture. Only two dimensions showed positivity above 50%. The highest dimension was “Teamwork within units” (57.4%) while the lowest positive mean score dimension was “Frequency of events reporting” (23.2%). That study recommended that patient safety needs to be incorporated into the education of health professionals [14]. The questionnaire used was adopted from the one designed for evaluation of the implementation of the WHO Patient safety curriculum for medical schools [13].

In this study, the mean age of the participants was about 28 years. They were particularly a young group of medical caregivers with about 40% of them graduated within the last 5 years, while in a study conducted in Alexandria university participants were in their mid-thirties [5]. The median years since fellowship completion was 13 years in a study by Berman et al. [15], which was an online survey, conducted on 353 surgeons addressing knowledge, attitudes, and perceptions surrounding the culture of patient safety. In a systematic review conducted by Brasaite et al., most of the health care professionals had many years of work experience (mean = 23.9 years) and their mean age was 46.7 years [12].

The current study included a multidisciplinary, relatively junior group of physicians, not like that by Berman et al. [15], but to some extent like that conducted in Alexandria University [5]. Only 20.3% of current study participants took courses in patient safety much less than those in Brasaite et al. where (54.4%) had received information during their continuing education about patient safety [16].

On a 5-point Likert scale, knowledge of these study participants which mainly centralized around error reporting was high in less than 40% of participants. In Berman et al. [15], Likert scale was dichotomized, and 60% of the participants stated that adverse event reporting improves patient safety, focusing on systems rather than individual accountability. In a study done on graduated medical students in Saudi Arabia, 42% of the participants rated their knowledge as good regarding the factors influencing patient safety, 37.3% regarding the different types of medical errors, and 28% regarding what should happen if an error is made [17]. That study stated that a study in UK concluded that medical students had little knowledge of how to report errors and also another multi-institutional survey demonstrated that knowledge levels are limited across different medical degrees and specialties. Also, findings of the study conducted by Brasaite et al. in three multi-disciplinary hospitals in Western Lithuania showed that health care professionals had low levels of safety knowledge [16] were in accordance with Oliveira et al.’s study conducted in a public university of Paraná, Brazil who stated that knowledge of patient safety among multi-professional residents was borderline satisfactory [18]. In a study conducted in Iran, 73% of students had negative opinions about “medical error reporting” [19]. On the other hand, in another study conducted in Italy, an unexpected high percentage of physicians (78.5%) believe that hospitals reporting medical errors voluntarily to a state agency reduces the number of medical errors [20].

Current study participants reported that their higher influence to improve patient safety was through talking about their own errors and filling in report forms. The majority in Almaramhy et al. study agreed to support those who make unintentional errors (76.0%) and 80.7% agreed not to blame their peers for their mistakes. In that study, participants did not recognize their active role in solving patient safety problems; however, they were willing to change practice to improve patient safety [17]. On the other hand, the surgeons in Berman et al.’s study declared that “The doctor bears ultimate responsibility for his/her patient's safety” [15].

Attaining high attitude toward patient safety among three quarters of current study participants is a positive finding—a higher percent than that reported by Almaramhy et al., around 50% [17]. This also goes in accordance with Brasaite et al.’s study which found that in general health care professionals had positive attitudes to patient safety [21]. Similarly, higher positive attitudes were reported in Flotta et al. in Italy ranging from 87 to 98% on different items among the participants [20]. As a related topic, Nabilou et al. stated that the respondents’ attitudes toward patient safety education were positive [19]. In the other studies, despite positive safety attitudes being reported, there are variations in how medical caregivers evaluated their attitudes in different dimensions regarding patient safety.

Higher positive attitude than gained knowledge toward patient safety pinpointed in this study is a common finding throughout related studies [12, 15, 17, 19]. Strikingly, there was not any correlation between knowledge and attitudes of participants in the current study. While in Brasaite et al., safety knowledge had significant positive low and medium correlations with attitudes [12].

In this study, the only association was between taking previous training courses in patient safety and caregivers’ perceived influence on patient safety. This goes in accordance with Berman et al. who did not find association between years since completion of fellowship and likelihood of feeling engaged in safety initiatives [15]. In the study conducted in Alexandria University [5], there was no statistically significant difference between perceptions of the participants in different work settings but their perceptions about patient safety decreased as their years of experience increased which is consistent with Brasaite et al. where participants who did not receive information about patient safety during their vocational and continuing education had a worse safety knowledge, and a positive correlation was found with the length of their work experience [16]. Flotta et al. concluded that the number of years elapsed since graduation was the only variable associated with the knowledge of evidence-based patient safety practices [20]. Moreover, a positive attitude was significantly predicted by a lower number of years elapsed since graduation. The relationships between students’ attitudes to patient safety and years of study, sex, and course were significant in Nabilou et al.’s study [19]. One last point is that, although responses were self-reported in an anonymous and confidential setting, yet we must take into consideration that using self-administered questionnaires may predispose participants to over- or under-report their attitudes. So, we may consider an even worst scenario than that depicted by the medical caregivers.

The WHO (2009) stated that one of the examples where further research is needed to reduce patient harm is poor knowledge [22]. Although the current study did not find a direct positive association between studied participants’ knowledge attitudes toward patient safety, other research suggests that medical care providers’ ability to deal with adverse events depends on their opportunities for learning. Attitudes related to patient safety issues were seen as positive among medical caregivers. It thus opens the door for open discussion of how to further develop their knowledge, discussing medical errors with colleagues, and reporting errors to supervisor followed by constructive feedback are ultimately important.

### Limitations of study

This study had some limitations including being a cross-sectional study that relied on self-reported knowledge, attitude, and influence; this is subject to reporting bias. Moreover, purposive sampling technique might hinder generalizability of results.

## Conclusion

Higher patient safety positive attitude than influence and knowledge was found in the current study. The main identified knowledge gaps were in ways of speaking up about errors, how to report errors, and the role of healthcare organization in error reporting. Physicians who took patient safety training courses reported higher influence score than those who did not. This raises attention to the importance of implementation of continuing patient safety education programs in terms of day courses, grand rounds, conferences, and meetings. It is recommended to repeat the study on a nationally representative sample of all health care workers.

## Data Availability

Authors report that the data supporting their findings can be publicly shared.

## Abbreviations

EMRO: East Mediterranean Regional Office
IOM: Institute of Medicine
MOHP: Ministry of Health and Population
REC: Research Ethical Committee
UK: United Kingdom
US: United States
WHO: World Health Organization
